# Prognostic Value of the Lung Immune Prognostic Index in Advanced Non-Small Cell Lung Cancer Treated with Nivolumab: A Real-World Analysis Beyond PD-L1 Expression

**DOI:** 10.3390/diagnostics16182890

**Published:** 2026-09-08

**Authors:** Alperen Akansel Çağlar, Aykut Özmen, Tuğrul Burak Genç, Anıl Yıldız, Özde Melisa Celayir, Shamkal Safarov, Yunus Avcı, Gökmen Umut Erdem, Nilüfer Bulut

**Affiliations:** Department of Medical Oncology, Başakşehir Çam and Sakura City Hospital, Istanbul 34110, Türkiye; draykutozmen@hotmail.com (A.Ö.); drtugrulburakgenc@gmail.com (T.B.G.); anilyildiz@live.com (A.Y.); melisacelayir84@gmail.com (Ö.M.C.); shamxalnn@gmail.com (S.S.); yunusavci2008@gmail.com (Y.A.); gokmenumut@hotmail.com (G.U.E.); ferlut@gmail.com (N.B.)

**Keywords:** lung immune prognostic index, non-small cell lung cancer, nivolumab, PD-L1 expression

## Abstract

**Background**: The Lung Immune Prognostic Index (LIPI), based on the derived neutrophil-to-lymphocyte ratio and lactate dehydrogenase, may reflect systemic inflammation and tumor-related metabolic burden. This study evaluated the prognostic value of pretreatment LIPI and its relationship with programmed death-ligand 1 (PD-L1) expression in patients with advanced non-small cell lung cancer (NSCLC) receiving nivolumab after first-line therapy. **Methods**: A total of 158 patients were included in this retrospective, single-center study. Patients were classified into LIPI 0, LIPI 1, or LIPI 2 groups according to their pretreatment LIPI scores. Progression-free survival (PFS) and overall survival (OS) were assessed using Kaplan–Meier analysis and multivariable Cox regression. The prognostic value of LIPI was also evaluated within PD-L1-negative and PD-L1-positive subgroups, and LIPI × PD-L1 interactions were explored. Harrell’s concordance index was used to examine whether adding LIPI improved model discrimination beyond clinical variables and PD-L1 expression. PD-L1 data were available for 145 patients. **Results**: Median PFS was 566, 340, and 109 days for the LIPI 0, LIPI 1, and LIPI 2 groups, respectively, while median OS was 841, 760, and 201 days, respectively; the differences were statistically significant for both outcomes (both *p* < 0.001). The objective response rate (ORR) was 55.6%, 36.5%, and 4.9%, whereas the disease control rate (DCR) was 75.9%, 65.1%, and 14.6% in the LIPI 0, LIPI 1, and LIPI 2 groups, respectively (both *p* < 0.001). In multivariable analyses, higher LIPI remained independently associated with shorter PFS and OS, with the strongest association observed in the LIPI 2 group. LIPI also differentiated survival outcomes within both PD-L1-negative and PD-L1-positive subgroups, although no significant LIPI × PD-L1 interaction was identified for PFS or OS. The addition of LIPI increased the C-index from 0.583 to 0.698 for PFS (ΔC-index, 0.115; *p* < 0.001) and from 0.588 to 0.697 for OS (ΔC-index, 0.109; *p* = 0.003). **Conclusions**: Pretreatment LIPI was independently associated with PFS and OS in patients with advanced NSCLC receiving nivolumab after first-line therapy. Its association remained after adjustment for available PD-L1 expression data, and its addition improved model discrimination. These findings suggest that LIPI may provide prognostic information complementary to PD-L1 expression and routinely available clinical factors.

## 1. Introduction

Lung cancer remains a major cause of cancer burden worldwide, not only because of its high incidence but also because it continues to be the leading contributor to cancer-related mortality. Non-small cell lung cancer (NSCLC) represents the predominant histological subtype, accounting for approximately 85% of lung cancer cases, and many patients are diagnosed when the disease has already reached an advanced stage [1,2,3]. In recent years, immune checkpoint inhibitors have changed the treatment landscape of advanced NSCLC and have provided clinically meaningful survival gains in selected patients [4]. However, the benefit obtained from immunotherapy is not uniform. Some patients achieve durable disease control, whereas others progress early despite treatment. This variability has made the search for reliable and easily applicable biomarkers an important issue in daily oncology practice [5].

Although tumor-based markers such as programmed death-ligand 1 (PD-L1) expression are widely used, they do not fully capture the complexity of treatment outcomes. PD-L1 expression provides clinically relevant information; however, intratumoral heterogeneity and differences in tissue sampling may affect its assessment and interpretation [6]. Clinical outcomes under immune checkpoint blockade are shaped not only by tumor-intrinsic factors but also by host-related conditions, including systemic inflammation, immune competence, and tumor burden. For this reason, blood-based prognostic indices have gained increasing attention. These indices are attractive because they are inexpensive, reproducible, and can be calculated from routine laboratory tests without the need for additional molecular procedures.

The Lung Immune Prognostic Index (LIPI) is one of the most commonly investigated inflammatory scores in this field. It is based on two parameters: the derived neutrophil-to-lymphocyte ratio (dNLR) and serum lactate dehydrogenase (LDH) level [7,8,9]. In clinical use, patients receive one point for dNLR ≥ 3 and one point for LDH above the upper limit of normal. According to the total score, they are classified into good, intermediate, and poor prognostic groups, corresponding to LIPI scores of 0, 1, and 2, respectively [4,10]. This simple structure makes LIPI particularly suitable for real-world settings, where complex biomarker testing may not always be available or may not reflect the patient’s overall inflammatory status.

The biological background of LIPI is closely linked to the interaction between cancer progression and host immunity. Neutrophils may support tumor growth through several mechanisms, including angiogenesis, invasion, metastatic dissemination, and suppression of effective antitumor immune responses. In contrast, lymphocytes are central components of immune surveillance and tumor cell elimination [7]. Therefore, a high dNLR may indicate a shift toward a more inflammatory and immunosuppressive systemic environment [11]. Consistent with this concept, elevated dNLR has been associated with poorer outcomes in patients treated with immune checkpoint inhibitors [12].

LDH provides another dimension of tumor biology within the LIPI score. Increased LDH levels may reflect higher tumor burden, hypoxia-driven metabolic reprogramming, and more aggressive disease behavior. Beyond being a marker of tumor load, LDH is also related to lactate accumulation in the tumor microenvironment. This metabolic change may impair T-cell function and weaken antitumor immune activity, which may partly explain why patients with elevated LDH tend to have less favorable outcomes during immunotherapy [13].

Several clinical studies have suggested that LIPI may have prognostic value in patients with advanced NSCLC treated with immune checkpoint inhibitors. In addition, post hoc analyses from large phase III immunotherapy trials, including the IMpower program, have shown that baseline LIPI is associated with both progression-free survival (PFS) and overall survival (OS). Nevertheless, most published studies have primarily evaluated LIPI in the overall treatment population, while evidence regarding its prognostic relevance within patient groups defined by PD-L1 expression remains comparatively limited [14,15].

This distinction is clinically relevant because PD-L1 expression and LIPI reflect different dimensions of cancer biology. PD-L1 is predominantly a tumor- and tumor microenvironment-based biomarker, whereas LIPI integrates systemic inflammatory activity and tumor-related metabolic burden. Therefore, the prognostic information provided by these markers may be complementary rather than overlapping. Determining whether LIPI can distinguish different clinical outcomes among patients with similar PD-L1 expression profiles may improve the clinical interpretation of both biomarkers.

This question is particularly relevant in previously treated patients receiving nivolumab, in whom PD-L1 expression alone may not fully account for the marked variability in clinical outcomes. Real-world data from this setting may help clarify whether a readily available blood-based index provides additional prognostic information beyond established tumor-based assessment.

## 2. Objective

This study aimed to examine the prognostic significance of pretreatment LIPI in patients with advanced NSCLC who received nivolumab after first-line systemic therapy. The primary objective was to evaluate its association with PFS and OS. Secondary objectives were to assess the prognostic relevance of PD-L1 expression, determine whether LIPI provided additional prognostic information within PD-L1-negative and PD-L1-positive patient groups, and evaluate whether LIPI retained its prognostic value after adjustment for PD-L1 expression and other relevant clinicopathological factors.

## 3. Materials and Methods

### 3.1. Study Design and Patient Population

This retrospective, single-center study evaluated patients with advanced NSCLC who were treated with nivolumab after progression on first-line systemic therapy. Patients who received nivolumab between October 2022 and October 2025 were screened through institutional electronic medical records.

Eligible patients were required to have histologically confirmed advanced NSCLC, an Eastern Cooperative Oncology Group performance status (ECOG PS) of 0 or 1, and sufficient clinical and laboratory data for LIPI calculation and survival analysis. In accordance with national reimbursement guidelines, all patients had previously received first-line platinum-based doublet chemotherapy without prior immune checkpoint inhibitors, with a minimum interval of 3 weeks between the last chemotherapy administration and the initiation of nivolumab. A total of 158 patients met these criteria and were included in the final cohort.

Patients with active or symptomatic brain metastases at the time of nivolumab initiation were excluded. However, patients with previously diagnosed brain metastases were eligible if they had received local treatment, such as surgery or radiotherapy, and had no clinical or radiological evidence of active symptomatic intracranial disease before starting nivolumab.

### 3.2. Data Collection

Baseline demographic, clinical, pathological, radiological, and laboratory data were obtained retrospectively from hospital records. Recorded variables included age, sex, smoking status, histological subtype, disease status, treatment line, metastatic sites, and PD-L1 expression status.

Laboratory values measured within 3 days prior to the first dose of nivolumab were used for analysis. These included white blood cell count, absolute neutrophil count, lymphocyte count, hemoglobin level, platelet count, serum LDH level and serum C-reactive protein (CRP) level. LDH measurements from hemolyzed blood samples were excluded to avoid potential analytical bias.

PD-L1 expression was reviewed from available pathology reports. Tumor PD-L1 status had been assessed using the Ventana SP263 immunohistochemistry assay (Ventana Medical Systems, Tucson, AZ, USA). PD-L1 data were available for 145 of the 158 patients. For the primary PD-L1 analyses, expression was categorized as 0%, 1–49%, and ≥50%. For subgroup analyses, patients were additionally classified as PD-L1-negative (0%) or PD-L1-positive (≥1%). Radiological records, including positron emission tomography–computed tomography (PET-CT), thoracic computed tomography (CT), and brain magnetic resonance imaging (MRI), were reviewed to determine disease extent and metastatic involvement.

### 3.3. LIPI Assessment

The LIPI was calculated before nivolumab treatment using two variables: dNLR and serum LDH level. The dNLR was calculated as follows:

- dNLR = neutrophil count/(white blood cell count − neutrophil count).

One point was assigned for each of the following factors:

- dNLR ≥ 3.
- LDH above the institutional upper limit of normal.

According to the total score, patients were categorized into three groups:

- LIPI 0: no risk factor.
- LIPI 1: one risk factor.
- LIPI 2: two risk factors.

**Figure 1:**
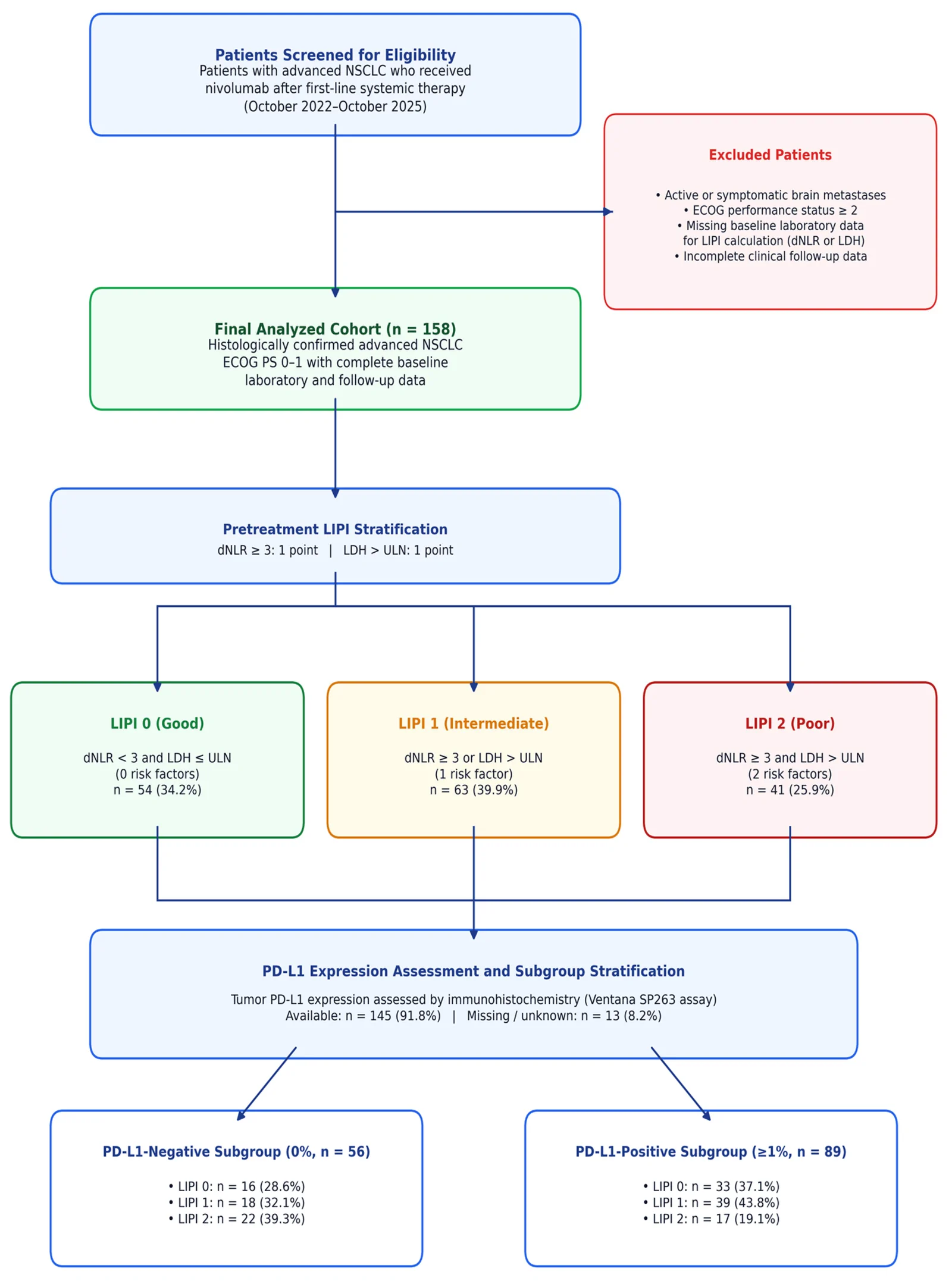
Patient selection flowchart. dNLR, derived neutrophil-to-lymphocyte ratio; ECOG PS, Eastern Cooperative Oncology Group performance status; LDH, lactate dehydrogenase; LIPI, Lung Immune Prognostic Index; NSCLC, non-small cell lung cancer; PD-L1, programmed death-ligand 1; ULN, upper limit of normal.

The overall study workflow, including patient selection, pretreatment LIPI assessment, and allocation according to PD-L1 status, is schematically presented in Figure 1.

### 3.4. Tumor Response Assessment

Tumor response was assessed retrospectively by reviewing all radiological evaluations performed during nivolumab treatment and classified according to Response Evaluation Criteria in Solid Tumors version 1.1 (RECIST v1.1). Response categories were defined as follows: complete response (CR), disappearance of all target and non-target lesions; partial response (PR), at least a 30% decrease in the sum of target lesion diameters relative to baseline; stable disease (SD), neither sufficient shrinkage to qualify as PR nor sufficient increase to qualify as progressive disease; and progressive disease (PD), at least a 20% increase in the sum of target lesion diameters from the nadir, or the appearance of new lesions. The best overall response was determined for each patient as the most favorable response recorded across all assessments during treatment. Objective response rate (ORR) was defined as the proportion of patients achieving a complete or partial response. Disease control rate (DCR) was defined as the proportion of patients achieving a complete response, partial response, or stable disease.

### 3.5. Survival Outcomes

PFS was defined as the interval from the first nivolumab administration to radiological or clinical disease progression or death from any cause, whichever occurred first. Tumor progression was assessed according to Response Evaluation Criteria in Solid Tumors version 1.1.

OS was calculated from the date of nivolumab initiation to death from any cause. Patients who were alive or progression-free at the last follow-up were censored on the date of their most recent clinical evaluation.

### 3.6. Ethics Statement

The study was approved by the Scientific Research Ethics Committee No. 2 of Başakşehir Çam and Sakura City Hospital (approval number: KAEK/06.05.2026.264). The study was conducted in accordance with the principles of the Declaration of Helsinki. Because of the retrospective design, the requirement for written informed consent was waived by the ethics committee.

### 3.7. Statistical Analysis

Statistical analyses were performed using IBM SPSS Statistics for Windows, version 25.0 (IBM Corp., Armonk, NY, USA) and R software (version 4.6.0, R Foundation for Statistical Computing, Vienna, Austria). Categorical variables were summarized as frequencies and percentages. Continuous variables were reported as mean ± standard deviation or median with minimum and maximum values, depending on the distribution of the data. Comparisons of ORR and DCR across the LIPI categories were performed using Fisher’s exact test.

The study was not based on a formal survival power calculation. All eligible patients who received nivolumab within the predefined study period and met the inclusion criteria were included. Therefore, the cohort should be regarded as a convenience sample reflecting real-world clinical practice.

The median follow-up duration was calculated using the reverse Kaplan–Meier method. Survival outcomes were estimated using the Kaplan–Meier method, and differences between LIPI groups were evaluated using the log-rank test. PFS and OS were also compared across PD-L1 expression categories. To evaluate the complementary prognostic role of LIPI, survival analyses according to LIPI category were performed separately in the PD-L1-negative and PD-L1-positive subgroups.

Multivariable Cox proportional hazards regression analyses were used to assess factors associated with PFS and OS. Covariates were selected a priori based on clinical and tumor-related factors commonly considered in advanced NSCLC and immunotherapy studies, rather than on univariable analysis results. The multivariable models included age, sex, PD-L1 expression, brain metastasis, liver metastasis, baseline CRP level, and LIPI category. In sensitivity analyses, the prognostic association of LIPI was additionally evaluated by modeling LIPI as an ordinal variable. The LIPI score was coded as 0, 1, and 2 and entered into the multivariable Cox regression models as a single ordinal covariate; the resulting hazard ratio represented the change in risk associated with each one-point increase in the LIPI score. To assess the incremental prognostic contribution of LIPI beyond clinical variables and PD-L1 expression, Harrell’s concordance index (C-index) was calculated for models including age, sex, PD-L1 expression, brain metastasis, liver metastasis, and CRP, with and without the addition of LIPI. Ninety-five percent confidence intervals (95% CI) for C-index values were estimated using bootstrap resampling with 1000 iterations. Differences in C-index between the nested models were formally compared using the compareC package in R software. In the multivariable Cox regression models, the proportional hazards assumption was assessed using the Grambsch–Therneau test based on Schoenfeld residuals. The assumption was tested separately for each covariate and globally for the overall model.

The potential modifying effect of PD-L1 status on the association between LIPI and survival was explored by including interaction terms between the three-category LIPI variable and binary PD-L1 status in the Cox regression models. For this analysis, PD-L1 status was defined as negative (0%) or positive (≥1%). The overall significance of the interaction was evaluated using the change in model likelihood after the two LIPI × PD-L1 interaction terms were added. This interaction analysis was considered exploratory.

Missing PD-L1 data were not imputed. Patients with missing PD-L1 expression were excluded from analyses including PD-L1 by listwise deletion; therefore, the effective sample size for these analyses was 145 patients. A *p* value of less than 0.05 was considered statistically significant.

## 4. Results

A total of 158 patients with advanced NSCLC were included in the final analysis. According to pretreatment LIPI score, 54 patients were classified as LIPI 0, 63 as LIPI 1, and 41 as LIPI 2. Baseline demographic and clinical characteristics are presented in Table 1.

Age, sex, ECOG PS, histological subtype, smoking status, PD-L1 expression, and the distributions of brain and bone metastases did not differ significantly across the three LIPI groups. Similarly, clinical stage and the number of metastatic sites were not significantly different between the groups. In contrast, significant between-group differences were observed in disease status, liver metastasis, adrenal metastasis, WBC count, and LDH levels. De novo disease and adrenal metastasis were more frequent in the LIPI 2 group, whereas liver metastasis was most frequent in the LIPI 0 group. Median WBC and LDH levels increased progressively from the LIPI 0 to the LIPI 2 group, while CRP levels did not differ significantly between the groups.

At the final data cutoff date of 1 July 2026, the median follow-up duration was 701 days (95% CI, 571.7–830.3), as estimated using the reverse Kaplan–Meier method. During follow-up, 130 PFS events, defined as disease progression or death, and 98 deaths were observed.

**Figure 2:**
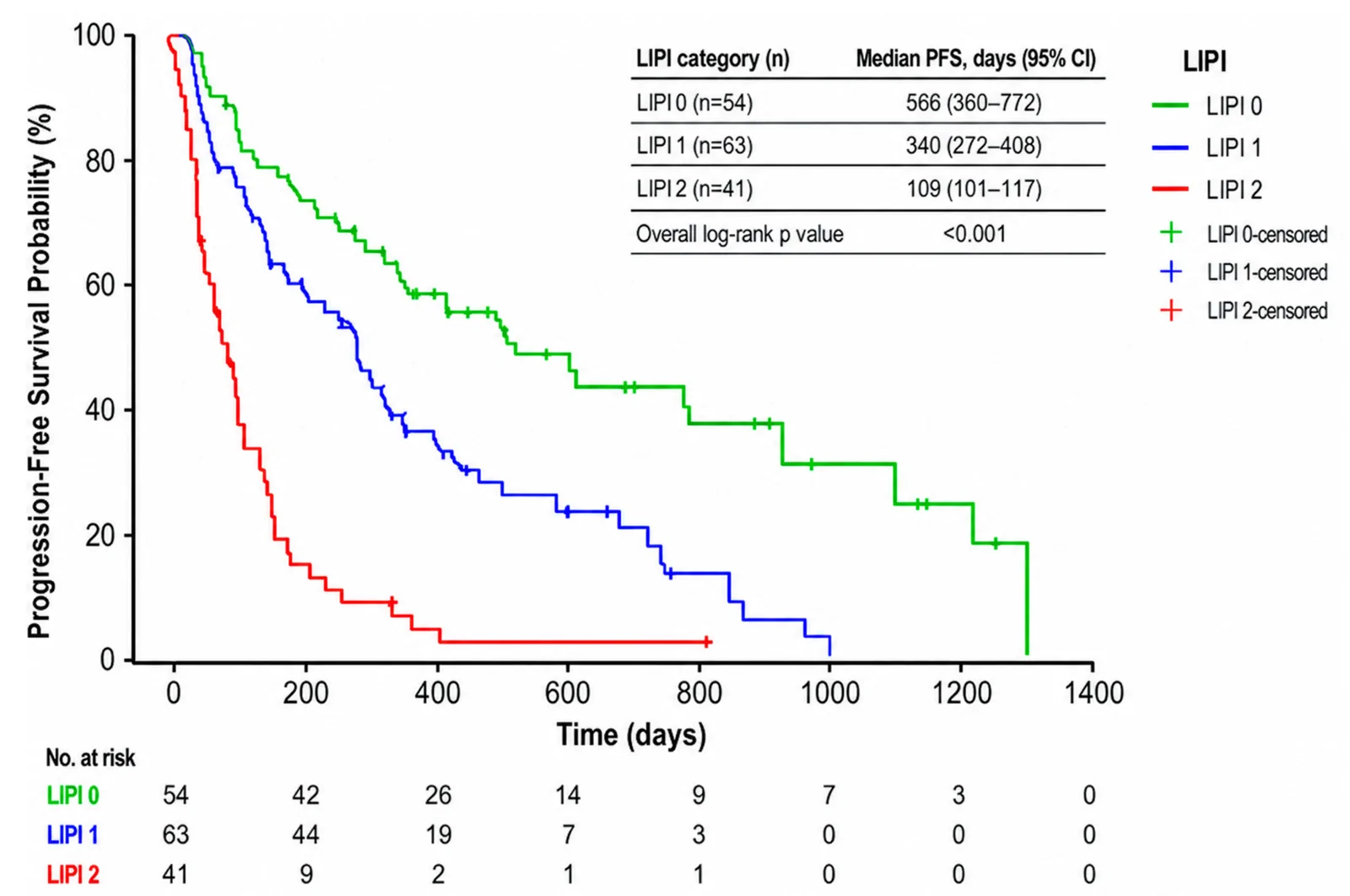
Kaplan–Meier curves for progression-free survival according to LIPI score.

Progression-free survival differed significantly across the three pretreatment LIPI categories. Median PFS was 566 days (95% CI, 360–772) in the LIPI 0 group, 340 days (95% CI, 272–408) in the LIPI 1 group, and 109 days (95% CI, 101–117) in the LIPI 2 group. The overall difference among the survival distributions was statistically significant (log-rank χ^2^ = 76.743; *p* < 0.001) (Figure 2). Patients in the LIPI 2 group had the shortest PFS.

**Figure 3:**
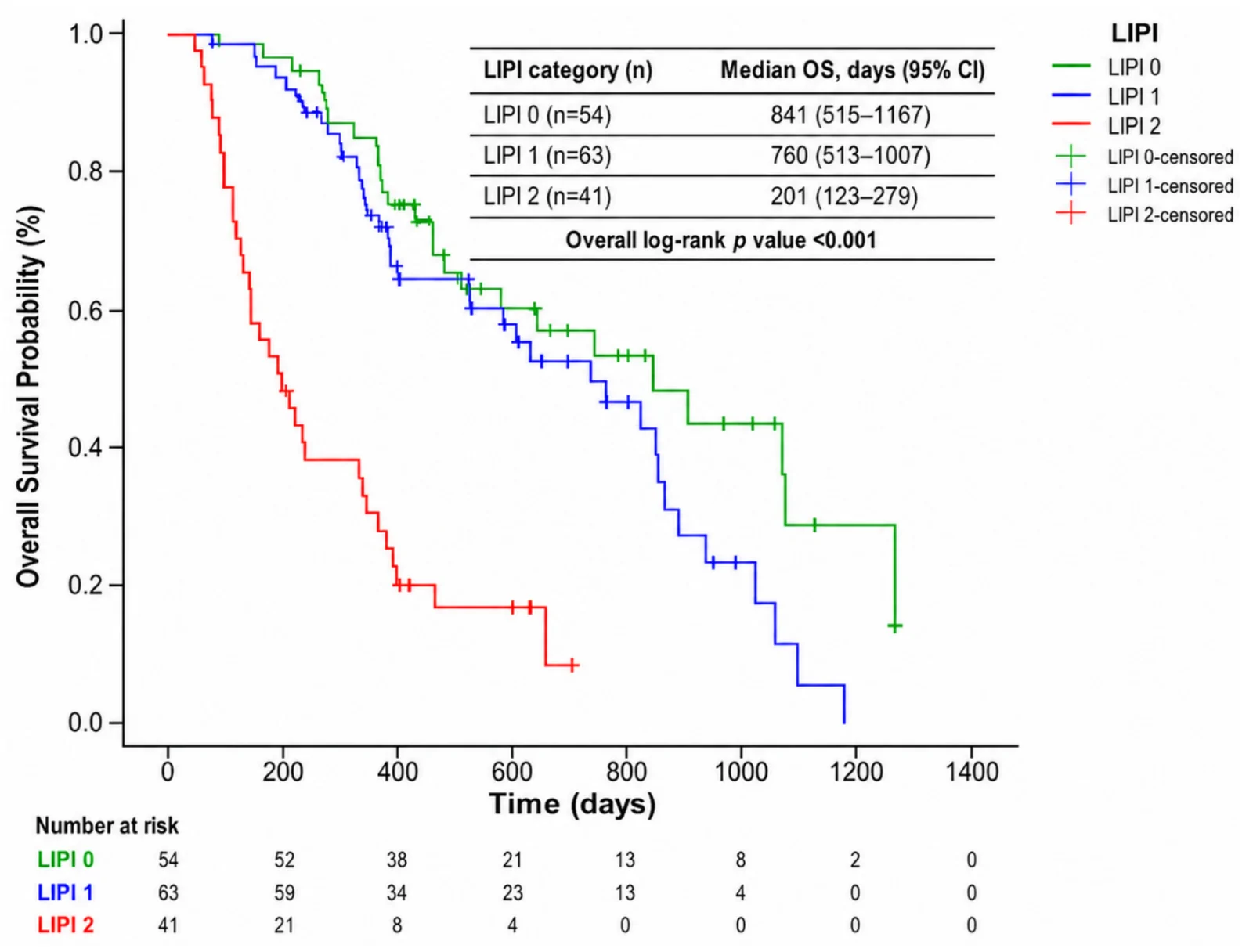
Kaplan–Meier curve for overall survival according to LIPI score.

Overall survival differed significantly according to pretreatment LIPI category. Median OS was 841 days (95% CI, 515–1167) in the LIPI 0 group, 760 days (95% CI, 513–1007) in the LIPI 1 group, and 201 days (95% CI, 123–279) in the LIPI 2 group. The overall difference among the three survival distributions was statistically significant (log-rank χ^2^ = 53.622; *p* < 0.001) (Figure 3). Patients classified as LIPI 2 had the shortest OS, whereas survival estimates were substantially longer in the LIPI 0 and LIPI 1 groups.

Tumor response outcomes also differed significantly according to LIPI score. The ORR was 55.6%, 36.5%, and 4.9% in the LIPI 0, LIPI 1, and LIPI 2 groups, respectively (*p* < 0.001). Similarly, the DCR was 75.9%, 65.1%, and 14.6% in the respective groups (*p* < 0.001). These findings are presented in Table 2.

**Figure 4:**
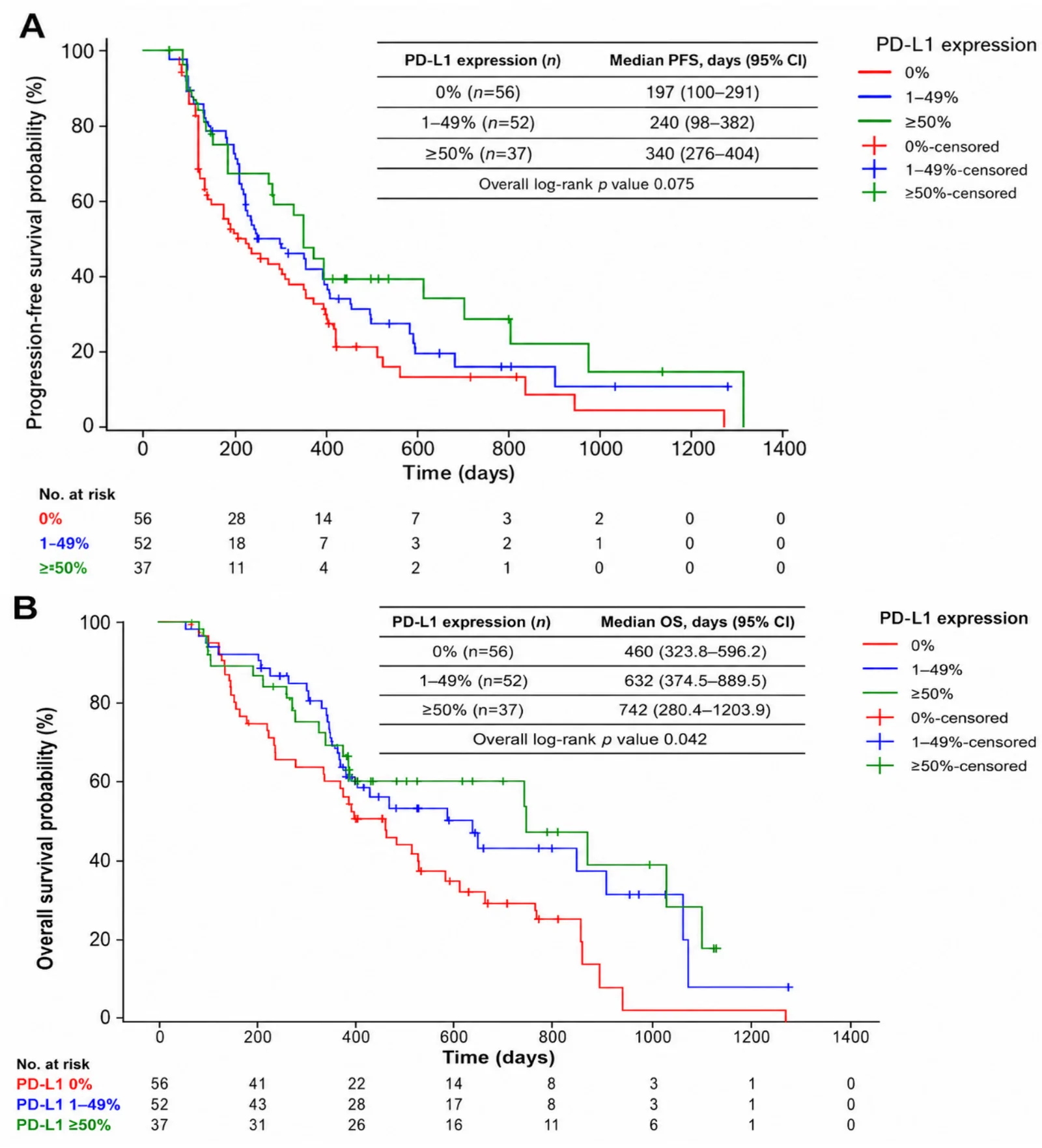
Kaplan–Meier curves for (**A**) progression-free survival and (**B**) overall survival according to PD-L1 expression levels.

Survival outcomes were evaluated according to PD-L1 expression levels, categorized as 0%, 1–49%, and ≥50%. Median progression-free survival was 197 days (95% CI, 103–291) in the PD-L1 0% group, 240 days (95% CI, 98–382) in the PD-L1 1–49% group, and 340 days (95% CI, 276–404) in the PD-L1 ≥ 50% group. Although median PFS increased numerically with higher PD-L1 expression, the difference among the groups did not reach statistical significance (log-rank *p* = 0.075; Figure 4A).

Median overall survival was 460 days (95% CI, 324–596) in the PD-L1 0% group, 632 days (95% CI, 374–890) in the PD-L1 1–49% group, and 742 days (95% CI, 280–1204) in the PD-L1 ≥ 50% group. A statistically significant difference in overall survival was observed among the PD-L1 groups (log-rank *p* = 0.042; Figure 4B), with longer survival observed at higher PD-L1 expression levels.

The prognostic value of LIPI was evaluated for PFS and OS in subgroups defined according to PD-L1 expression status. Because of the limited number of patients in some PD-L1 categories, patients were analyzed in two subgroups: PD-L1-negative (0%) and PD-L1-positive (≥1%).

**Figure 5:**
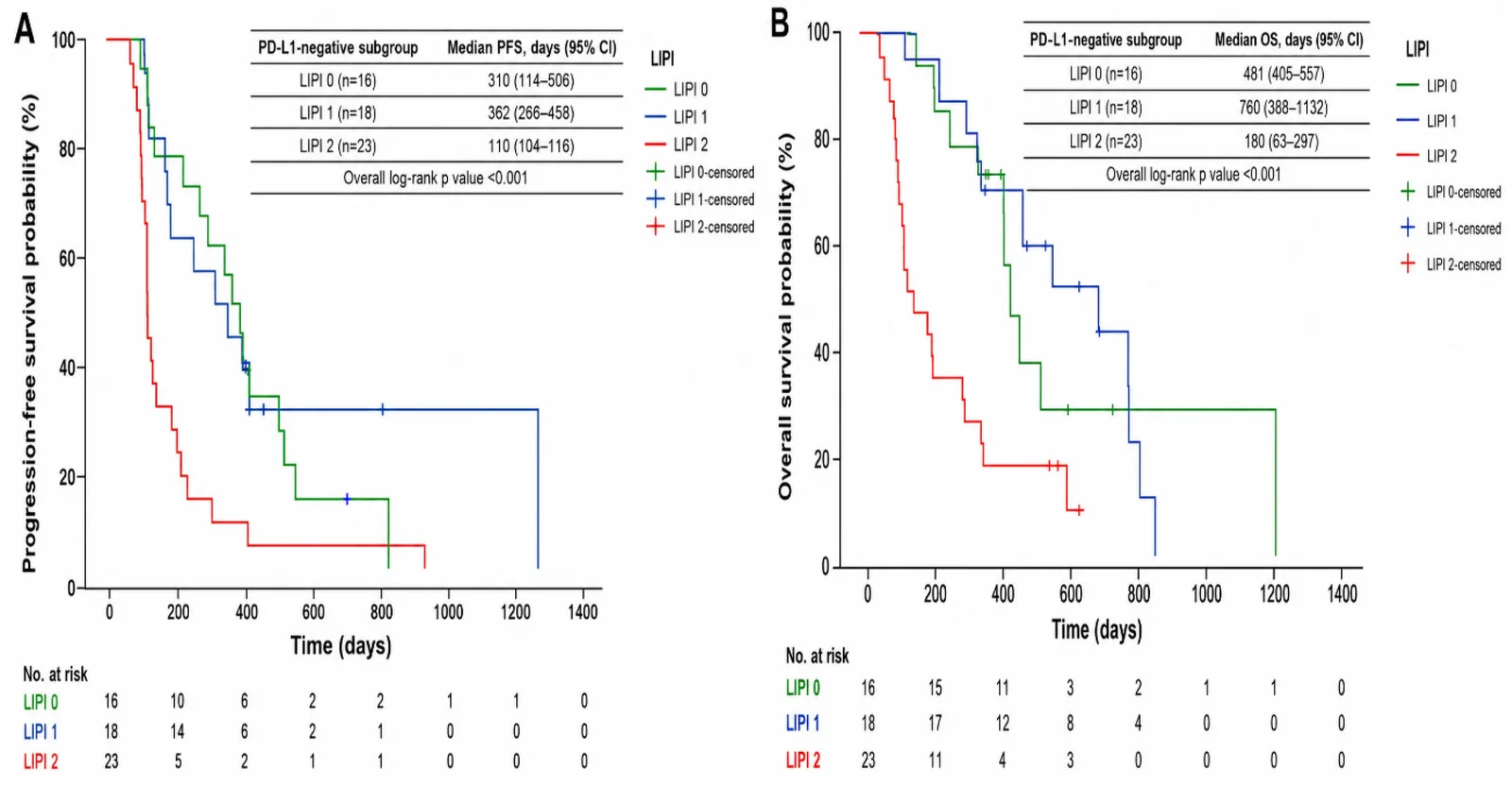
Kaplan–Meier curves for (**A**) progression-free survival and (**B**) overall survival according to LIPI in the PD-L1-negative subgroup.

In the PD-L1-negative subgroup, PFS differed across the LIPI categories. Median PFS was 310 days (95% CI, 114–506) in the LIPI 0 group, 362 days (95% CI, 266–458) in the LIPI 1 group, and 110 days (95% CI, 104–116) in the LIPI 2 group (log-rank *p* < 0.001; Figure 5A). The shortest median PFS was observed in the LIPI 2 group.

OS also varied across the LIPI categories in the PD-L1-negative subgroup. Median OS was 481 days (95% CI, 405–557) in the LIPI 0 group, 760 days (95% CI, 388–1132) in the LIPI 1 group, and 180 days (95% CI, 63–297) in the LIPI 2 group (log-rank *p* < 0.001; Figure 5B). The shortest median OS was observed in the LIPI 2 group.

**Figure 6:**
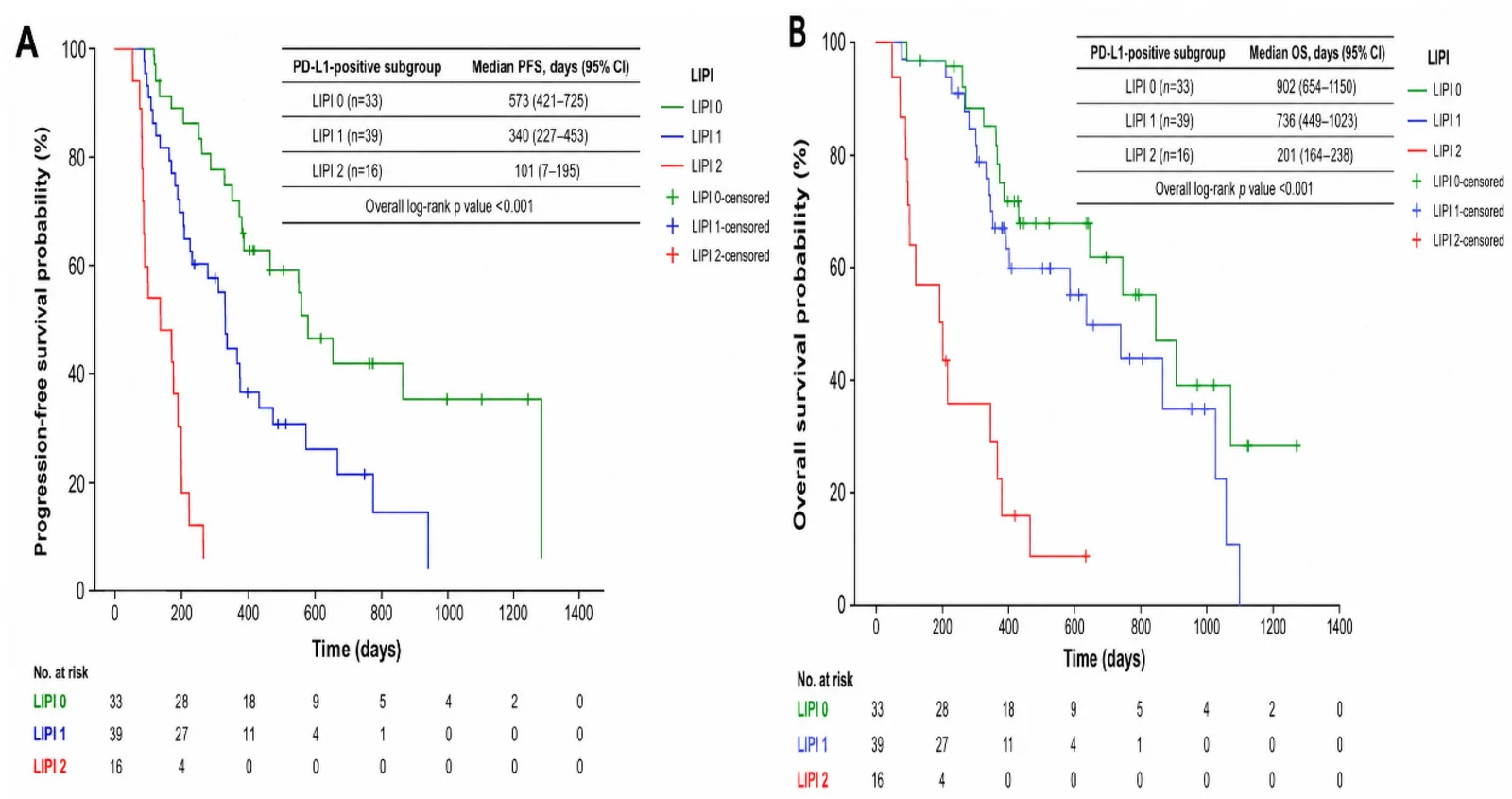
Kaplan–Meier curves for (**A**) progression-free survival and (**B**) overall survival according to LIPI in the PD-L1-positive subgroup.

In the PD-L1-positive subgroup, PFS varied across the LIPI categories. Median PFS was 573 days (95% CI, 421–725) in the LIPI 0 group, 340 days (95% CI, 227–453) in the LIPI 1 group, and 101 days (95% CI, 7–195) in the LIPI 2 group (log-rank *p* < 0.001; Figure 6A). The LIPI 2 group showed a shorter PFS than the LIPI 0 and LIPI 1 groups.

OS also varied across the LIPI categories in the PD-L1-positive subgroup. Median OS was 902 days (95% CI, 654–1150) in the LIPI 0 group, 736 days (95% CI, 449–1023) in the LIPI 1 group, and 201 days (95% CI, 164–238) in the LIPI 2 group (log-rank *p* < 0.001; Figure 6B). A shorter OS was observed in the LIPI 2 group compared with the LIPI 0 and LIPI 1 groups.

Additionally, to evaluate whether the prognostic effect of LIPI varied according to PD-L1 status, interaction terms between the three-category LIPI variable and dichotomized PD-L1 status (0% vs. ≥1%) were included in the multivariable Cox models. No statistically significant interaction was observed for either PFS (*p* for interaction = 0.371) or OS (*p* for interaction = 0.654). These findings suggest that the prognostic effect of LIPI was consistent regardless of PD-L1 status. When PD-L1 status was further stratified into three categories (0%, 1–49%, and ≥50%) and interaction terms were re-tested accordingly, no statistically significant interaction was observed for either PFS (*p* for interaction = 0.797) or OS (*p* for interaction = 0.373), consistent with the primary analysis. However, these subgroup analyses should be interpreted with caution given the small number of patients in certain strata.

Cox regression analyses were performed for PFS and OS to determine whether the prognostic effect of LIPI was independent of PD-L1 expression and key clinical factors potentially associated with survival in advanced NSCLC. Thirteen patients with missing PD-L1 data were excluded from models that included PD-L1, and missing values were not imputed.

Univariable analysis showed that LIPI and CRP were significantly associated with PFS, whereas the overall effect of the three-category PD-L1 variable did not reach statistical significance. In the multivariable Cox regression analysis, LIPI remained independently associated with PFS when evaluated together with age, sex, PD-L1 expression, brain metastasis, liver metastasis, and baseline CRP. Compared with the LIPI 0 group, the risk of progression or death was approximately twofold higher in the LIPI 1 group (HR, 2.03; 95% CI, 1.25–3.30; *p* = 0.004) and approximately eightfold higher in the LIPI 2 group (HR, 7.85; 95% CI, 4.37–14.10; *p* < 0.001). PD-L1 expression was not independently associated with PFS in the multivariable model (overall *p* = 0.739), and none of the other covariates reached statistical significance (Table 3).

In the univariable analysis for OS, LIPI was significantly associated with survival (overall *p* < 0.001); the risk of death was markedly higher in the LIPI 2 group than in the LIPI 0 group (HR, 5.879; 95% CI, 3.376–10.238; *p* < 0.001). PD-L1 expression was also significantly associated with OS in the univariable analysis (overall *p* = 0.045); patients with PD-L1 expression ≥ 50% had a lower risk of death than those with PD-L1 expression of 0% (HR, 0.545; 95% CI, 0.315–0.945; *p* = 0.031).

In the multivariable Cox regression analysis, LIPI remained independently associated with OS when evaluated together with age, sex, PD-L1 expression, brain metastasis, liver metastasis, and baseline CRP. The risk of death was approximately sevenfold higher in the LIPI 2 group than in the LIPI 0 group (HR, 6.790; 95% CI, 3.608–12.780; *p* < 0.001). In contrast, PD-L1 expression was not independently associated with OS in the multivariable model (overall *p* = 0.627), and none of the other covariates reached statistical significance (Table 4). In the assessment of the proportional hazards assumption using Schoenfeld residuals, no significant departure from the assumption was observed for the PFS model (global *p* = 0.304). The global test for the OS model was also not statistically significant (global *p* = 0.164); however, a result suggestive of a departure from proportionality was obtained for the LIPI variable (*p* = 0.024).

In sensitivity analyses, modeling LIPI as a single ordinal covariate yielded results consistent with the primary categorical models. Each one-point increase in the pretreatment LIPI score was independently associated with a higher risk of progression or death (adjusted HR, 2.788; 95% CI, 2.055–3.783; *p* < 0.001) and a higher risk of death (adjusted HR, 2.666; 95% CI, 1.905–3.730; *p* < 0.001).

The addition of the LIPI score improved model discrimination for both survival outcomes when evaluated using Harrell’s concordance index and nested model comparison via the compareC test. For PFS, the C-index increased from 0.583 (95% CI 0.543–0.661) in the base clinical model to 0.698 (95% CI 0.660–0.761) with the inclusion of LIPI (ΔC-index = 0.115; *p* < 0.001). For OS, the C-index increased from 0.588 (95% CI 0.551–0.678) in the base model to 0.697 (95% CI 0.653–0.774) after incorporating LIPI (ΔC-index = 0.109; *p* = 0.003) (Table 5).

## 5. Discussion

This real-world study was conducted to evaluate the prognostic value of pretreatment LIPI in patients with advanced NSCLC who received nivolumab after first-line systemic therapy. In addition, we examined how the prognostic contribution of LIPI varied across PD-L1 expression levels and whether it provided information beyond PD-L1 assessment alone. In our cohort, LIPI was significantly associated with both PFS and OS, with higher scores corresponding to poorer survival outcomes. The clearest and most consistent prognostic separation for both endpoints was observed in the LIPI 2 group. These findings are in line with previous clinical studies, real-world series, post hoc analyses of phase III trials, and meta-analyses showing the prognostic relevance of LIPI in patients with advanced NSCLC treated with immune checkpoint inhibitors [5,7,14,15,16,17,18,19].

The difference in OS between the LIPI 0 and LIPI 1 groups was less pronounced than the separation observed for LIPI 2. In the multivariable OS model, the risk of death was numerically higher in the LIPI 1 group but did not reach statistical significance, whereas a marked increase in risk was observed in the LIPI 2 group. Accordingly, the most robust and consistent prognostic signal in our study appears to be associated with the LIPI 2 category. Findings for the LIPI 1 group should therefore be interpreted more cautiously and warrant confirmation in larger cohorts.

Nevertheless, the pronounced prognostic separation observed in the LIPI 2 group is consistent with the shorter PFS and OS reported for the LIPI 2 group in the multicenter real-world study by Ruiz-Bañobre et al. among patients receiving nivolumab [16]. Although direct comparisons of risk estimates are not appropriate because of differences in patient characteristics, treatment settings, and analytical approaches across studies, the sensitivity analysis modeling LIPI as an ordinal covariate showed that each one-point increase in the LIPI score was associated with an approximately 2.8-fold higher adjusted risk of progression or death and an approximately 2.7-fold higher adjusted risk of death for PFS and OS, respectively. These findings support the interpretation that the prognostic association was not solely attributable to the predefined three-category classification.

In addition to survival outcomes, tumor response rates showed a consistent gradient across LIPI categories. Both ORR and DCR declined progressively with higher LIPI scores, with the most pronounced difference observed in the LIPI 2 group. These findings are consistent with the directional pattern reported by Mezquita et al., in which response rates decreased markedly with worsening LIPI in patients receiving immune checkpoint inhibitors for advanced NSCLC, and with real-world nivolumab-specific data reported by Ruiz-Bañobre et al. [15,16]. The response rates observed in our cohort are consistent with these published series and suggest that the adverse prognostic profile associated with LIPI 2 extends beyond survival endpoints to encompass tumor response. The higher ORR observed in our overall cohort (34.8%) and particularly in the LIPI 0 subgroup (55.6%) compared with rates reported in the second-line nivolumab CheckMate 017 and CheckMate 057 trials should be interpreted cautiously, as this disparity may be influenced by retrospective design, non-protocolized imaging assessments, and institutional cohort selection [20,21]. Furthermore, given the absence of a non-immunotherapy control group, these findings should be interpreted in a prognostic rather than a predictive context.

Similarly to the findings of Ruiz-Bañobre et al., one of the first large-scale studies to evaluate LIPI in patients receiving nivolumab after first-line therapy, PD-L1 expression levels have generally not been reported or PD-L1 subgroup-specific LIPI analyses have not been adequately performed in the existing series in this setting [16]. As a result, how LIPI and PD-L1 together shape the prognostic landscape has not been sufficiently clarified in these studies. In the present study, PD-L1 data were available for the majority of patients, allowing the prognostic value of LIPI to be examined separately within PD-L1-negative and PD-L1-positive subgroups and enabling model discrimination analyses in which LIPI was evaluated alongside PD-L1. These analyses suggest that LIPI may provide additional prognostic information beyond PD-L1 expression, an aspect that has not been systematically addressed in prior nivolumab-specific real-world series.

In the Kaplan–Meier analyses according to PD-L1 expression, increasing expression levels were accompanied by numerical improvements in both PFS and OS; however, the difference between groups reached statistical significance only for OS. The literature regarding the association between PD-L1 expression and survival in patients receiving nivolumab in the second or later treatment line remains heterogeneous. In CheckMate 057, higher PD-L1 expression was associated with more favorable outcomes with nivolumab, whereas in CheckMate 017 the clinical activity of nivolumab was observed irrespective of PD-L1 expression level [20,21]. Real-world studies have likewise reported a trend toward longer survival with higher PD-L1 expression, although this association has not been consistently confirmed across all series [22]. Variability in PD-L1 assessment related to intratumoral and intertumoral heterogeneity, sampling characteristics, and the immunohistochemical method used may also contribute to differences across studies [20,23,24,25]. Thus, the OS difference observed in our cohort, which was not mirrored in PFS, suggests that the prognostic contribution of PD-L1 may vary among previously treated patients receiving nivolumab.

One of the notable findings of this study was that LIPI continued to distinguish survival outcomes within both the PD-L1-negative and PD-L1-positive subgroups. Among PD-L1-negative patients, the shortest PFS and OS were observed in the LIPI 2 group. A similar pattern was seen in the PD-L1-positive subgroup, in which patients with LIPI 2 again had the least favorable survival outcomes. These results indicate that clinically meaningful heterogeneity may persist among patients with the same PD-L1 expression status and may be further characterized by LIPI.

The shorter PFS and OS observed among PD-L1-positive patients with a LIPI score of 2 suggest that PD-L1 positivity alone does not necessarily translate into a more favorable clinical course. Conversely, the relatively longer survival observed in PD-L1-negative patients with lower LIPI scores supports the view that systemic inflammatory burden, as reflected by LIPI, may influence outcomes independently of tumor PD-L1 status. Taken together, these observations suggest that LIPI and PD-L1 may reflect distinct, yet potentially complementary, dimensions of prognosis in this setting.

Nevertheless, the significant survival differences observed in subgroup analyses should not be interpreted as evidence that PD-L1 status modifies the prognostic effect of LIPI. No statistically significant LIPI × PD-L1 interaction was identified for either PFS or OS. This indicates that there was insufficient evidence to conclude that the association between LIPI and survival differed materially between PD-L1-negative and PD-L1-positive patients. The limited number of patients in some subgroup categories may also have reduced the statistical power of the interaction analysis and made modest effect modification more difficult to detect. Overall, the subgroup and interaction findings suggest that LIPI retains prognostic relevance in both PD-L1 groups, although a PD-L1-dependent difference in the strength of this association could not be demonstrated.

In the Cox regression analyses, univariable analysis showed a lower risk of death in patients with PD-L1 expression ≥ 50% than in those with PD-L1 expression of 0%, whereas higher CRP levels were associated with shorter PFS. However, when PD-L1 expression, CRP, and other clinical variables were included in multivariable models together with LIPI, these associations were no longer statistically significant. In contrast, LIPI remained independently associated with both PFS and OS. The increased risks observed in the LIPI 1 and LIPI 2 groups persisted for PFS, while the strongest and statistically significant association with OS was again observed in the LIPI 2 group. The departure from proportionality observed for LIPI in the OS model suggests that the association between LIPI and OS may not have remained completely constant throughout follow-up. This may be related to a higher concentration of events in the LIPI 2 group during the early period, with the difference in risk between groups diminishing over time. Therefore, the OS hazard ratio for LIPI should be interpreted cautiously. Beyond its independent association in multivariable Cox models, incorporating LIPI into a model containing clinical variables and PD-L1 expression improved discrimination, suggesting that LIPI may provide incremental prognostic information in this setting.

These findings suggest that the prognostic association of LIPI may partly reflect its ability to capture information related to both systemic inflammation and disease burden [5,14,16,26]. Blood-based inflammatory and metabolic markers have previously been associated with survival in NSCLC and other solid tumors, and baseline characteristics of this kind have also been reported to provide prognostic information in patients receiving immunotherapy [27,28,29,30,31]. Biologically, an elevated dNLR may reflect a protumoral and immunosuppressive inflammatory state, in which neutrophil-mediated mechanisms may attenuate effective antitumor T-cell responses, whereas a relative reduction in lymphocyte-mediated immune surveillance may limit the development of a sustained antitumor immune response [32]. Elevated LDH may indicate increased tumor burden and tumor-associated metabolic dysregulation; hypoxic and lactate-rich conditions may further impair the proliferation and effector functions of antitumor immune cells, potentially reducing the benefit derived from immune checkpoint inhibition [33]. Collectively, these mechanisms may contribute to an immunosuppressive tumor–host environment associated with unfavorable outcomes during immune checkpoint inhibitor therapy. The persistence of the association between LIPI and survival after adjustment for PD-L1, CRP, brain metastases, liver metastases, age, and sex supports the view that this score may reflect a broader combination of clinical and biological features relevant to outcome. At the same time, the loss of statistical significance for PD-L1 and CRP in multivariable models should not be interpreted as evidence that these variables lack clinical relevance. Their observed associations may overlap with other prognostic characteristics, and cohort size, event numbers, and correlations among covariates may also have contributed to the attenuation of these effects.

Within this context, the independent prognostic value of LIPI supports its potential role as a complementary tool for baseline risk stratification in patients with advanced NSCLC receiving nivolumab. Whether its integration alongside PD-L1 expression can meaningfully improve clinical decision-making warrants further evaluation in larger and prospective studies.

## 6. Limitations

This study has several limitations. The retrospective, single-center design and the relatively small number of patients in some subgroups may limit the generalizability of the findings and reduce the precision of the subgroup, interaction, and multivariable analyses. In addition, the lack of protocol-defined timing for radiological assessments and follow-up may have introduced information and ascertainment bias. Therefore, the absolute PFS and OS estimates, particularly within the LIPI subgroups, should be interpreted cautiously when extrapolated to other patient populations. PD-L1 expression data were not available for all patients; accordingly, PD-L1-based analyses were restricted to patients with available data, and missing values were not imputed. The influence of unmeasured confounding factors that could not be reliably assessed retrospectively, including baseline infection status, corticosteroid or antibiotic use, overall disease burden, and residual effects of prior chemotherapy on bone marrow function and inflammatory parameters despite the three-week interval before nivolumab initiation, and post-progression treatments, cannot be completely excluded.

Because the study did not include a non-immunotherapy control group, the findings support the prognostic value of LIPI but do not establish its predictive value for nivolumab-specific treatment benefit. Furthermore, because the cohort was limited to patients receiving nivolumab monotherapy after first-line treatment, this study could not determine whether the prognostic association of LIPI is consistent across other immune checkpoint inhibitors or varies according to the specific agent. Finally, the absence of an independent external validation cohort limits the robustness of the findings.

## 7. Conclusions

In conclusion, pretreatment LIPI was independently associated with progression-free survival and overall survival in patients with advanced NSCLC receiving nivolumab after first-line therapy. This association remained after adjustment for available PD-L1 expression data, suggesting that LIPI may provide complementary prognostic information beyond tumor-based biomarkers. Given its simplicity, low cost, and reliance on routinely available laboratory parameters, LIPI may represent a potential prognostic stratification tool in this clinical setting; however, its clinical utility requires confirmation in larger, prospective, and externally validated cohorts.

## Figures and Tables

**Table 1:** Baseline Patient Characteristics According to LIPI Score.

Variable	LIPI 0 (*n* = 54)	LIPI 1 (*n* = 63)	LIPI 2 (*n* = 41)	*p* Value
**Age, mean**	65	64	63	0.646
**Sex, *n* (%)**				0.518
Male	48 (88.9)	53 (84.1)	33 (80.5)	
Female	6 (11.1)	10 (15.9)	8 (19.5)	
**ECOG PS, *n* (%)**				0.872
0	10 (18.5%)	11 (17.5%)	8 (19.5%)	
1	39 (72.2%)	47 (74.6%)	26 (63.4%)	
Unknown	5 (9.3%)	5 (7.9%)	7 (17.1%)	
**Histology, *n* (%)**				0.919
Non-squamous	34 (63.0)	42 (66.7)	26 (63.4)	
Squamous	19 (35.2)	20 (31.7)	13 (31.7)	
Unknown	1 (1.9)	1 (1.6)	2 (4.9)	
**Smoking status, *n* (%)**				0.505
Never smoker	6 (11.1)	5 (7.9)	6 (14.6)	
Smoker	25 (46.3)	22 (34.9)	21 (51.2)	
Ex-smoker	19 (35.2)	27 (42.9)	13 (31.7)	
Unknown	4 (7.4)	9 (14.3)	1 (2.4)	
**Disease status, *n* (%)**				0.039
De novo	30 (55.6)	45 (71.4)	33 (80.5)	
Recurrent	23 (42.6)	18 (28.6)	8 (19.5)	
Unknown	1 (1.9)	0	0	
**TNM-based clinical stage at nivolumab initiation, *n* (%)**				0.123 *
Stage IIIC	2 (3.7)	4 (6.3)	4 (9.8)	
Stage IVA	28 (51.9)	37 (58.7)	14 (34.1)	
Stage IVB	24 (44.4)	22 (34.9)	23 (56.1)	
**Number of metastatic** **sites, *n* (%)**				0.060 *
0	2 (3.7)	4 (6.3)	4 (9.8)	
1	19 (35.2)	22 (34.9)	8 (19.5)	
2	18 (33.3)	29 (46.0)	14 (34.1)	
≥3	15 (27.8)	8 (12.7)	15 (36.6)	
**PD-L1 group, *n* (%)**				0.052
0%	16 (29.6)	18 (28.6)	22 (53.7)	
1–49%	16 (29.6)	25 (39.7)	11 (26.8)	
≥50%	17 (31.5)	14 (22.2)	6 (14.6)	
Unknown	5 (9.3)	6 (9.5)	2 (4.9)	
**Bone metastasis, *n* (%)**				0.931
No	31 (57.4)	34 (54.0)	23 (56.1)	
Yes	23 (42.6)	29 (46.0)	18 (43.9)	
**Liver metastasis, *n* (%)**				0.012
No	37 (68.5)	57 (90.5)	33 (80.5)	
Yes	17 (31.5)	6 (9.5)	8 (19.5)	
**Brain metastasis, *n* (%)**				0.956
No	37 (68.5)	43 (68.3)	27 (65.9)	
Yes	17 (31.5)	20 (31.7)	14 (34.1)	
**Adrenal metastasis, *n* (%)**				0.017
No	45 (83.3)	49 (77.8)	24 (58.5)	
Yes	9 (16.7)	14 (22.2)	17 (41.5)	
**WBC, ×10^9^/L, median**	6.40 (5.38–7.99)	8.13 (6.32–10.10)	10.56 (8.99–11.79)	<0.001
**LDH, U/L, median**	173.0 (155.8–181.5)	235.0 (168.0–286.0)	295.0 (259.5–350.5)	<0.001
**CRP, mg/L, median**	15.95 (6.10–37.00)	26.00 (7.70–59.00)	22.00 (11.70–90.00)	0.138

Data are presented as mean, median, or n (%), as appropriate. Comparisons were performed using one-way analysis of variance, the Kruskal–Wallis test, or Pearson’s chi-square test, as appropriate. * *p* values were calculated using Fisher’s exact test. Unknown values were excluded from relevant analyses. A two-sided *p* value < 0.05 was considered statistically significant. CRP, C-reactive protein; ECOG PS, Eastern Cooperative Oncology Group performance status; LIPI, Lung Immune Prognostic Index; PD-L1, programmed death-ligand 1; TNM, tumor-node-metastasis; WBC, white blood cell; LDH, lactate dehydrogenase.

**Table 2:** Objective response rate and disease control rate according to LIPI score.

LIPI Score	ORR, *n* (%)	DCR, *n* (%)
0 (***n*** = 54)	30 (55.6)	41 (75.9)
1 (***n*** = 63)	23 (36.5)	41 (65.1)
2 (***n*** = 41)	2 (4.9)	6 (14.6)
**Total (*n* = 158)**	55 (34.8)	88 (55.7)
***p* value**	<0.001	<0.001

*p* values were calculated using Fisher’s exact test. DCR, disease control rate; LIPI, Lung Immune Prognostic Index; ORR, objective response rate.

**Table 3:** Univariable and Multivariable Cox Regression Analyses for Progression-Free Survival.

Variable (Comparison)	Univariable HR (95% CI)	*p*	Multivariable HR (95% CI)	*p*
**Age (per year)**	0.993 (0.971–1.014)	0.504	0.990 (0.969–1.012)	0.374
**Sex (male vs. female)**	0.773 (0.482–1.241)	0.287	0.878 (0.514–1.499)	0.634
**PD-L1 (overall)**	—	0.079	—	0.739
PD-L1 1–49% (vs. 0%)	0.736 (0.488–1.111)	0.145	1.012 (0.653–1.569)	0.956
PD-L1 ≥ 50% (vs. 0%)	0.593 (0.369–0.954)	0.031	0.841 (0.508–1.394)	0.503
**Brain metastasis (yes vs. no)**	0.859 (0.595–1.240)	0.417	1.276 (0.859–1.897)	0.228
**Liver metastasis (yes vs. no)**	0.819 (0.535–1.254)	0.358	1.461 (0.907–2.352)	0.119
**CRP (per mg/L)**	1.004 (1.001–1.006)	0.010	0.998 (0.995–1.001)	0.251
**LIPI (overall)**	—	<0.001	—	<0.001
LIPI 1 (vs. 0)	2.034 (1.306–3.168)	0.002	2.032 (1.249–3.304)	0.004
LIPI 2 (vs. 0)	7.002 (4.292–11.422)	<0.001	7.845 (4.365–14.098)	<0.001

CI, confidence interval; CRP, C-reactive protein; HR, hazard ratio; LIPI, Lung Immune Prognostic Index; PD-L1, programmed death-ligand 1. “—” indicates that a hazard ratio was not applicable for the overall categorical comparison. A two-sided *p* value < 0.05 was considered statistically significant.

**Table 4:** Univariable and Multivariable Cox Regression Analyses for Overall Survival.

Variable (Comparison)	Univariable HR (95% CI)	*p*	Multivariable HR (95% CI)	*p*
**Age (per year)**	0.999 (0.974–1.023)	0.914	0.996 (0.971–1.021)	0.740
**Sex (male vs. female)**	0.946 (0.526–1.702)	0.854	1.440 (0.764–2.713)	0.260
**PD-L1 (overall)**	—	0.045	—	0.627
**PD-L1 1–49% (vs. 0%)**	0.624 (0.388–1.005)	0.053	0.843 (0.516–1.379)	0.496
**PD-L1 ≥ 50% (vs. 0%)**	0.545 (0.315–0.945)	0.031	0.771 (0.434–1.369)	0.374
**Brain metastasis (yes vs. no)**	1.300 (0.860–1.966)	0.213	1.288 (0.820–2.024)	0.272
**Liver metastasis (yes vs. no)**	1.328 (0.823–2.142)	0.245	1.496 (0.863–2.594)	0.151
**CRP (per mg/L)**	1.003 (1.000–1.006)	0.064	0.999 (0.995–1.003)	0.535
**LIPI (overall)**	—	<0.001	—	<0.001
LIPI 1 (vs. 0)	1.553 (0.933–2.585)	0.090	1.654 (0.930–2.942)	0.087
LIPI 2 (vs. 0)	5.879 (3.376–10.238)	<0.001	6.790 (3.608–12.780)	<0.001

CI, confidence interval; CRP, C-reactive protein; HR, hazard ratio; LIPI, Lung Immune Prognostic Index; PD-L1, programmed death-ligand 1. “—” indicates that a hazard ratio was not applicable for the overall categorical comparison. A *p* value < 0.05 was considered statistically significant.

**Table 5:** Incremental prognostic contribution of LIPI to model discrimination.

Outcome	Base Model C-Index (95% CI)	Base Model + LIPI C-Index (95% CI)	ΔC-Index	*p* Value
**PFS**	0.583 (0.543–0.661)	0.698 (0.660–0.761)	0.115	<0.001
**OS**	0.588 (0.551–0.678)	0.697 (0.653–0.774)	0.109	0.003

The base model included age, sex, PD-L1 expression, brain metastasis, liver metastasis, and baseline CRP. After excluding patients with missing PD-L1 data, the effective sample size was *n* = 145. Ninety-five percent confidence intervals for the C-index estimates were obtained using 1000 bootstrap resamples, and differences between nested models were assessed using the compareC method. CI, confidence interval; C-index, concordance index; CRP, C-reactive protein; LIPI, Lung Immune Prognostic Index; OS, overall survival; PD-L1, programmed death-ligand 1; PFS, progression-free survival.

## Data Availability

The data presented in this study are available upon reasonable request from the corresponding author. The data are not publicly available due to privacy and ethical restrictions.

## Abbreviations

CI	Confidence Interval
C-index	Concordance Index
CRP	C-Reactive Protein
CT	Computed Tomography
DCR	Disease Control Rate
dNLR	Derived Neutrophil-to-Lymphocyte Ratio
ECOG	Eastern Cooperative Oncology Group
HR	Hazard Ratio
LDH	Lactate Dehydrogenase
LIPI	Lung Immune Prognostic Index
MRI	Magnetic Resonance Imaging
ORR	Objective Response Rate
OS	Overall Survival
PD-L1	Programmed Death-Ligand 1
PET-CT	Positron Emission Tomography–Computed Tomography
PFS	Progression-Free Survival
SPSS	Statistical Package for the Social Sciences
